# How does temporomandibular dysfunction affect neck awareness, cervical sensorimotor performance, and pain sensitivity?

**DOI:** 10.1590/1806-9282.20251522

**Published:** 2026-06-29

**Authors:** Nagihan Acet, Murat Esmer, Sena Begen, Mustafa Karahan

**Affiliations:** 1 Atılım University, Faculty of Health Sciences, Department of Physiotherapy and Rehabilitation – Ankara, Türkiye.; 2 Gazi University, Faculty of Health Sciences, Department of Physiotherapy and Rehabilitation – Ankara, Türkiye.; 3 Special Dünyapark Hospital – Diyarbakır, Türkiye.

**Keywords:** Temporomandibular joint, Awareness, Proprioception, Pain perception, Pain threshold, Neck pain

## Abstract

**OBJECTIVE:**

The temporomandibular joint is anatomically and neurologically associated with the cervical spine. The aim of this study was to investigate the impact of temporomandibular disorder on neck awareness, cervical sensorimotor performance, and pain sensitivity, including pressure pain threshold, pressure pain tolerance, and temporal summation.

**METHODS:**

In this observational, cross-sectional study, the diagnostic criteria for temporomandibular disorders were used to evaluate temporomandibular disorder. Trial registered as NCT06558318. Participants were divided into two groups based on temporomandibular disorder presence: a temporomandibular disorder group (n=25) and an asymptomatic control group (n=25). Neck awareness was evaluated using the Fremantle Neck Awareness Questionnaire, while cervical sensorimotor performance was assessed using the "joint position error test" for both the global cervical region and the isolated upper cervical region, measured separately with a cervical range of motion device. Pain pressure threshold, pain pressure tolerance, and temporal summation were measured using a mechanical pressure algometer.

**RESULTS:**

No significant differences were found between the temporomandibular disorder and control group in pain-related parameters (p>0.05). Neck awareness was significantly impaired in the temporomandibular disorder group (p<0.001), although the joint position error tests showed no significant differences (p>0.05).

**CONCLUSION:**

The presence of temporomandibular disorder did not affect pain sensitivity in the cervical region. While sensorimotor performance was not impaired in either the global or the isolated upper cervical region, neck awareness was affected in the temporomandibular disorder group. This suggests that neck awareness in patients with temporomandibular disorder should be considered in clinical evaluation and management.

## INTRODUCTION

The term "temporomandibular disorder" (TMD) refers to structural and functional abnormalities related to the chewing muscles and/or the temporomandibular joint (TMJ), with or without clinical signs and symptoms. Its prevalence ranges from 28 to 88%, and it is common among young adults aged 20–40, impairing quality of life^1^. Early diagnosis of TMD is critical for preventing advanced problems that may arise later^2^. Therefore, the early identification of the effects of TMD is crucial for determining preventive treatment approaches.

The temporomandibular region is anatomically connected to the cervical area through ligaments and muscles, leading to the idea that postural problems may influence the development of TMD. The position and movement of the TMJ can influence cervical spine alignment. Similarly, changes in the cervical region, particularly in the upper cervical spine, can affect mandibular position, potentially leading to TMD^3^.

Moreover, the trigeminal nerve and upper cervical nerves converge at the trigeminal nucleus and the cervical trigeminal complex in the brainstem^4^. This neurological connection can lead to an interaction between pain sensations in both the jaw and neck regions^4^.

Although the anatomical and neurological connections between the TMJ and cervical region are well established, studies examining how these regions are perceived and represented at a perceptual level remain limited. Neck awareness (NA) is an aspect of somatic awareness that reflects how individuals perceive, represent, and direct attention toward their cervical region. Previous research has shown that such perceptual processes can be disrupted in various musculoskeletal pain conditions. For example, patients with Complex Regional Pain Syndrome and low back pain exhibit disturbances in the mental representation of their bodies, including difficulties mentally rotating the affected body part, feelings of detachment or non-ownership of the painful region, and challenges in locating the painful limb in space^5^. Additionally, chronic pain conditions have been associated with alterations in the location and size of somatosensory cortical representations corresponding to the affected body part^6^. These findings suggest that a bidirectional relationship may exist between pain and perceptual body representation. Indeed, experimental manipulations that visually alter body awareness have been shown to increase or decrease pain intensity^7^. Taken together, these findings imply that changes in NA in the context of TMD may not solely arise as a consequence of pain; rather, disturbances in perceptual representation may themselves influence pain modulation, indicating a potentially bidirectional interaction^8^. Despite this relevance, studies investigating NA in individuals with TMD are scarce, and this remains an important gap in the literature.

Proprioception is the sense that allows the perception of "the position of body parts in space"^9^. Although proprioceptive errors, particularly in the extremities, have been linked to an increased risk of injury, the research examining the connection between TMD and cervical sensorimotor performance (CSP) is quite limited^10,11^. Current studies have primarily been performed in individuals with severe pain. However, pain may not be the main complaint in the early or moderate stage of TMD, and symptoms of TMD do not always show up as noticeable jaw discomfort, particularly in younger populations. In this population, pain can be absent or remain at a mild intensity that is easily overlooked or dismissed, contributing to delayed recognition of TMD-related problems.

Another important aspect to consider in TMD is cervical pain sensitivity. The literature includes studies evaluating pressure pain threshold and tolerance in the cervical region of patients with TMD^10,12–14^. However, no studies have assessed temporal summation in the cervical region in TMD.

This study aimed to investigate the impact of TMD on NA, CSP, and cervical pain sensitivity.

### Study design

This cross-sectional and observational study was written using the STROBE (Strengthening the Reporting of Observational Studies in Epidemiology) checklist. It was approved by the "Atılım" University Non-Interventional Research Ethics Committee (Number: 604.01.02-161/ date: 24.01.2024). The study was conducted per the principles stated in the Declaration of Helsinki. After providing information about the study to the participants, those who agreed to participate were included, between 19.08.2024 and 01.10.2024, after gaining their written consent. Registration of clinical trials is as follows: NCT06558318.

### Participants

Young individuals aged between 18 and 25 were included in the study. Exclusion criteria were a history of cervical spine injuries or disorders, chronic pain conditions, diagnosed musculoskeletal disorders, prior neck or spine surgeries, or neurological or psychiatric conditions affecting proprioception or pain perception.

In the initial phase, a total of 178 individuals who participated in the TMD awareness event organized at the Faculty of Health Sciences, Atılım University, were evaluated using the Fonseca Anamnestic Index. The Fonseca Anamnestic Index is a validated, reliable tool that evaluates the existence of TMD and its severity based on the signs and symptoms of the disorder^15^. It contains 10 items to which respondents answer with one of three possibilities: yes (10 points), sometimes (5 points), or no (0 points). The final score is obtained by adding up all answers. It ranges from a minimum score of 0 (no signs of TMD) to a maximum score of 100 (severe TMD), with the following classifications: 0–15 (no TMD), 20–45 (mild TMD), 50–65 (moderate TMD), and 70–100 (severe TMD)^15^. The Turkish version was used in the current study^16^. It has a Cronbach alpha of 0.82, an intraclass correlation coefficient of 0.93, a cut-off point of >35 points, a sensitivity of 83.33%, and a specificity of 77.97%.

Subsequently, participants were invited to the physiotherapy and rehabilitation department laboratory for further assessments. Among these, 60 individuals who agreed to undergo additional evaluations were reassessed using the Diagnostic Criteria for Temporomandibular Disorders (DC/TMD)^17^. During this process, 10 individuals were excluded from the study for various reasons. As a result, a total of 50 young individuals aged 22.34±1.33 years were included in the study.

Table 1 presents the distribution of DC/TMD Axis I diagnoses in the TMD group. According to the DC/TMD criteria, each participant received a single Axis I diagnosis. In Group I (Myofascial Pain), 52% of participants were diagnosed with myofascial pain without limited opening (I a), and 4% were diagnosed with myofascial pain with limited opening (I b). In Group II (Disc Displacement), 24% of participants presented with disc displacement with reduction (II a), while 12% were diagnosed with disc displacement without reduction (II b). In Group III (Arthralgia), 8% of participants were diagnosed with arthralgia (III a).

**Table 1:** Diagnostic criteria/temporomandibular disorder axis I diagnostic distribution in the temporomandibular disorder group.

DC/TMD group	Subtype	Description	n	%
Group I—Myofascial Pain	I a	Without a limited opening	13	52%
	I b	With limited opening	1	4%
Group II—Disc displacement	II a	With reduction	6	24%
	II b	Without reduction	2	12%
Group III—Arthralgia	III a	Arthralgia	2	8%
Total			25	100%

DC/TMD: Diagnostic Criteria for Temporomandibular Disorders; TMD: temporomandibular dysfunction; n: number of participants.

### Sample size

The sample size calculation was conducted using G*POWER software (Version 3.1.9.7; Faul, Erdfelder, Lang, & Buchner, 2007). In the post power analysis, the number of participants to be included in the study was calculated as 42 when the type I error was 0.05, the power (1-β error) was 0.80, and the estimated effect size (Cohen's d) was 0.5. However, a total of 50 participants have been included to reduce bias and increase the accuracy of the findings.

### Outcome measurements

The participants’ age (years), height (m), and body weight (kg) were recorded. Body mass index (BMI) values (kg/m²) were calculated by dividing body weight by the square of height.

The pain-related parameters, including pain threshold, pain tolerance, temporal summation, and proprioception, were assessed in both groups by a researcher who did not know which group the participants were in. Thus, the blinding of the evaluator was ensured.

### Assessment of neck awareness

NA was assessed using the Fremantle Neck Awareness Questionnaire^18^. The Fremantle Neck Awareness Questionnaire typically consists of nine items, each rated on a Likert scale from 0 to 4. The scale ranges from 0 to 36 points. Higher scores on this scale indicate impaired NA^18^.

### Assessment of cervical sensorimotor performance

It was assessed using two different methods to capture different components of cervical joint position sense. First, joint position error was measured for the total cervical region to assess global CSP. Second, an additional assessment was conducted for the isolated upper cervical region using a separate testing protocol. Both evaluations were performed with a cervical range of motion (CROM) device to quantify repositioning accuracy and detect subtle deficits specific to each cervical segment.

Assessment of CSP for global cervical region: The cervical position error test was conducted using a CROM device in two directions: right rotation and left rotation, while participants sat on a chair with back support and feet in full contact with the floor^19^. Firstly, the maximum cervical range of motion (ROM) was determined for both directions. The midpoint of each participant's maximum ROM was defined as the "target position." With their eyes closed, participants’ heads were passively moved to the target position and held there for 3 s, and then returned to the neutral position. Participants were then asked to actively move their head back to the same target position, and this movement was repeated six times. The differences between the target position and the achieved position were recorded and averaged. To maintain standardization across participants, the Joint Position Error test was always performed first in right rotation and then in left rotation^19^.

Assessment of CSP for isolated upper cervical region: The cervical flexion-rotation test and a CROM device were used to evaluate joint position sense^19^. While the participant was in a sitting position and feet in full contact with the floor, the cervical region was passively brought into full flexion, thereby locking the lower cervical vertebrae. Firstly, the maximum ROM for cervical rotation was then determined for both directions in this position. The midpoint of each participant's maximum ROM was defined as the "target position." With their eyes closed, participants’ heads were passively moved to the target position while in full flexion, held there for 3 s, and then returned to the neutral position. Participants were then asked to actively move their head back to the same target position, and this movement was repeated six times. The differences between the target position and the achieved position were recorded and averaged.

### Assessment of pain sensitivity

In this study, pain was assessed using three complementary methods: pressure pain threshold, pressure pain tolerance, and temporal summation. A pressure algometer (Baseline Force Gauge Model 12–0304; Baseline, NY, USA) was used to assess all pain assessment procedures. All procedures were performed bilaterally by the same experienced therapist while the individual sat in a chair with back support and feet fully contacting the floor to ensure standardization of the procedure.

#### Assessment of pressure pain threshold

The pressure was applied perpendicularly and bilaterally using the algometer to different points (over the TMJ—anterior to the tragus—2 cm lateral to the C2 spinous processes, at the midpoint of the upper part of the trapezius muscle) at a rate of approximately 3 N/s over an area of 0.5 cm². The participant was asked to report the moment they first felt discomfort as the pressure was applied, and the pressure value at this moment was recorded from the device as the "pressure pain threshold" and noted on the test protocol. A second measurement was taken at each region after an interval, and the average of the two measurements was noted.

#### Assessment of pain tolerance

Following the pain threshold measurement, participants were given a 5-min rest period. Pain tolerance was then evaluated at the same anatomical points. Participants were asked to endure the applied pressure up to the maximum level they could tolerate and to report when they could no longer withstand the pressure. The final pressure value displayed on the device at that point was recorded as the pressure pain tolerance. Participants were instructed to endure the applied pressure for as long as tolerable and to indicate when it became intolerable. The pressure value at this moment was recorded from the device as the "pain tolerance" and noted on the test protocol.

#### Assessment of temporal summation

Ten minutes after measuring the pressure pain threshold assessment, a temporal summation (TS) assessment was conducted bilaterally at the same points (2 cm lateral to C2 and C6, at the midpoint of the upper trapezius). For each test site, mechanical pressure was applied at an intensity equal to the participant's pressure pain threshold value—that is, the minimum pressure at which discomfort was first perceived. The therapist applied 10 consecutive pressure stimuli, each maintained for approximately 0.5 s, with an interstimulus interval of 1 s, which represents the standard timing required to elicit wind-up–like pain responses in TS testing. Participants rated the pain intensity of the first and tenth stimulus, using the Numerical Pain Scale (NPS; 0=no pain, 10=worst imaginable pain). The TS value was calculated by subtracting the pain rating of the first stimulus from that of the tenth^20,21^.

### Statistical analysis

The analyses were conducted using International Business Machines Statistical Package for the Social Sciences (IBM SPSS) Statistics (IBM SPSS Statistics for Windows, Version 27. Armonk, NY: IBM Corp.). Frequencies (number, percentage) were reported for categorical variables, and descriptive statistics (mean, standard deviation) were provided for numerical variables. The normality of numerical variables across groups was tested using the Shapiro-Wilk test, visual inspection (histograms and Q–Q plots), skewness, and kurtosis values. An independent samples t-test was used to compare the two groups.

Because multiple pain-sensitivity outcomes were evaluated—specifically, pressure pain threshold, pain tolerance, and TS measured at C2, C6, and the upper trapezius—these variables were considered a single family of related tests. To control the family-wise Type I error rate, the Holm–Bonferroni correction was applied across the pain-sensitivity p-values.

Cohen's d effect sizes and 95%CIs were calculated for all parameters between-group comparisons.

## RESULTS

The study was completed with a total of 50 participants, 25 of whom were in the TMD group and 25 in the control group. The demographic characteristics of the participants in both groups are shown in Table 2. The groups were well-matched in terms of age, height, weight, and BMI, with no statistically significant differences observed between them (p>0.05). When compared in terms of the presence of TMD, there was a significant difference between the two groups (p<0.001). The mean Fonseca score for the TMD group was 40.6±4.63, compared to 4.6±4.98 for the control group.

**Table 2:** Demographic characteristics of the participants.

	TMD group (n=25)		Control group (n=25)		Mean difference	p-value	95%CI	Cohen's d
	$\bar{\text{X}}$ ± SD	Min–max	$\bar{\text{X}}$ ± SD	Min–max				
Age (year)	22.04±1.33	19–25	22.6±1.29	20–25	0.35	0.139	−0.48 to 1.19	0.29
Height (cm)	1.68±0.08	1.53–1.92	1.72±0.08	1.54–1.86	0.04	0.153	−0.13 to −0.09	0.53
Weight (kg)	68.8±13.74	47–100	68.12±11.93	47–84	0.17	0.719	−8.36 to 8.72	0.01
BMI (kg/m^2^)	23.3±3.66	16.85–32.28	22.77±2.74	18.59–27.08	−1.09	0.557	−3.49 to 1.31	0.32
FAI scores	40.6±4.63	35–45	4.6±4.98	0–15	−36	**<0.001** *	−38.73 to −33.26	7.48

TMD: temporomandibular dysfunction; n: number of participants; BMI: body mass index; FAI: Fonseca Anamnestic Index; $\bar{\text{X}}$ : Mean; SD: standard deviation; CI: confidence interval; cm: centimeter; kg: kilogram; kg/m^2^: kilogram/meter^2^. The bold values indicate statistically significant results (p<0.05).

* Indicates statistically significant results (p<0.05).

Comparisons of groups in terms of pain sensitivity, NA, and CSP were presented in Table 3. The results showed a significant difference in the Fremantle Neck Awareness Questionnaire scores between the groups, suggesting that NA may be affected by TMD (p<0.001). However, no significant differences were observed in other parameters, including joint position error tests, pain threshold, pain tolerance, and TS after Holm–Bonferroni correction.

**Table 3:** The comparisons of pain sensitivity, neck awareness, and sensorimotor performance between groups.

			TMD group (n=25)		Control group (n=25)		Mean difference	p-value	95%CI	Cohen's d
			$\bar{\text{X}}$ ± SD	Min–max	$\bar{\text{X}}$ ± SD	Min–max				
Pain threshold										
	TMJ		2.77±1.14	1.4–6.25	2.60±0.95	1.20–5.05	−0.170	0.571	−0.71 to 0.39	−0.161
	C2		3.22±1.24	1.9–6.5	3.23±1.09	1.95–5.75	0.006	0.986	−0.66 to 0.67	0.005
	C6		3.93±1.61	2–9.35	3.87±1.18	2.25–6.85	−0.062	0.898	−0.87 to 0.74	−0.044
	Midpoint of the upper trapezius		4.36±1.8	2.4–9.7	4.27±1.92	1.5–8.5	−0.094	0.859	−1.15 to 0.96	−0.050
Pain tolerance										
	C2		5.04±2.76	2.2–11	5.72±2.36	2.75–11	0.682	0.354	−0.78 to 2.14	0.265
	C6		6.06±2.41	3.15–11	6.46±2.15	3.5–11	0.394	0.546	−0.9 to 1.69	0.172
	Midpoint of the upper trapezius		6.68±2.27	3.65–11	7.32±2.65	2.75–11	0.64	0.365	−0.76 to 2.04	0.258
Temporal summation										
	C2		3.33±1.75	1.45–9.4	3.12±1.27	1.3–5.7	−0.21	0.621	−1.18 to 0.65	−0.141
	C6		4.22±1.96	1.8–9.1	3.75±1.44	1.55–6.55	−0.472	0.338	−1.45 to 0.5	−0.274
	Midpoint of the upper trapezius		4.41±2.33	1.35–10.05	4.26±1.87	1. 6–7. 5	−0.154	0.798	−1.35 to 1.05	−0.073
Neck awareness										
	FNAQ scores		10.04±7.28	0–22	2±3.97	0–17	-8.04	**<0.001** *	-11.37 to −4.67	−1.370
Cervical sensorimotor performance										
	Global cervical region									
		Right rotation	3.47±4.38	0.33–21.67	3.5±3.67	0.33–13.67	0.43	0.981	-0.27 to 2.32	0.159
		Left rotation	2.9±3.31	0–13.83	3.93±3.05	0.5–11.67	-1.56	0.261	-0.78 to 2.83	−0.429
	Isolated upper cervical region*									
		Right rotation	2.56±2.08	0–8.33	3.03±2.76	0–9.5	-0.47	0.504	-0.92 to 1.85	0.190
		Left rotation	2.89±2.25	0–6.67	3.86±3.44	0–12.83	-0.97	0.243	-1.68 to 2.62	0.335

TMD: temporomandibular dysfunction; n: number of participants; $\bar{\text{X}}$ : FNAQ: Fremantle Neck Awareness Questionnaire; Mean; SD: standard deviation; CI: confidence interval; TMJ: temporomandibular joint.

* Isolated Upper Cervical Region was assessed using the cervical flexion-rotation test.

The bold values indicate statistically significant results (p<0.05).

## DISCUSSION

This study aimed to investigate the impact of TMD on NA, CSP, and cervical pain-related parameters. No significant differences were found between the groups in CSP, pressure pain threshold, pressure pain tolerance, and TS. However, NA was significantly impaired in the TMD group.

In the present study, individuals in the TMD group exhibited marked disturbances in NA. This difference was not only statistically significant but also demonstrated a large effect size, indicating a substantial magnitude of impairment.

Neuroscientific models indicate that somatic awareness does not arise from a single sensory modality but from the integration of visceral sensations, proprioceptive–vestibular inputs, and fine-touch information within the central nervous system^1^. These sensory inputs are processed across multiple neural networks, including the brainstem, thalamus, parietal and temporal cortices, insula, and prefrontal regions, thereby forming one's perception of body position, movement, and regional bodily integrity^1^. Consequently, conditions affecting the orofacial and cervical regions, such as TMD, may alter these sensory inputs, leading to impaired somatic awareness and changes in regional body representation. This framework provides a neurobiological rationale for the disturbances in NA observed among individuals with TMD in the present study.

Body awareness and postural awareness have previously been shown to be directly associated with musculoskeletal pain and emotional well-being^2^. Habitual teeth-clenching behavior is considered one of the primary contributing factors to the development of TMD^3,4^. Such habitual postural patterns observed in the TMD group may therefore be linked to lower NA and poorer postural habits.

It is known that TMD criteria do not fully correspond with MRI findings and that false-positive rates may be high. This suggests that TMD cannot be explained solely by structural changes and that clinical symptoms may also be related to sensorimotor and neurophysiological mechanisms^22^. In another study, it was emphasized that proprioceptive and sensorimotor alterations in individuals with TMD are not limited to peripheral structural impairments and that central processing mechanisms may also be involved in the process. In our findings, the impairment of NA may have been caused by changes at the level of body perception and cortical representation^23^.

It has been suggested that TMD may influence postural control through the jaw sensorimotor system^5^. When considered together with existing literature, the present results imply that improving awareness may exert direct or indirect beneficial effects on musculoskeletal pain and emotional status in individuals with TMD^6^.

The presence of marked impairments in body awareness among individuals with TMD highlights the potential etiological relevance of NA in TMD and suggests that reductions in awareness may emerge prior to measurable sensorimotor deficits. Future studies should investigate how changes in NA are triggered by TMD, how they progress over time, and what implications they hold for clinical assessment and early intervention.

Although somatic awareness was clearly altered in individuals with TMD in the present study, this pattern did not extend to CSP. Evidence examining the relationship between TMD and CSP is still limited, and existing studies have reported inconsistent findings^10,11,13^. However, our findings related to the joint position error test differ from those reported in the literature.

The present study was the first to evaluate CSP for the isolated upper cervical region in addition to CSP for the global cervical region. Previous research has demonstrated that dysfunction in the upper cervical spine may influence TMD through shared muscular attachments, trigeminocervical convergence, and sensorimotor interactions. These interactions suggest that the upper cervical region may play an important role in the sensorimotor mechanisms associated with TMD. However, there was no difference between the groups in terms of CSP for both the global and isolated upper cervical regions. We believe that this situation may be attributable to the fact that the participants in our study were young adults and generally exhibited low levels of pain.

Another cervical region parameter addressed in the present study was cervical pain sensitivity. The analysis revealed that TMD had no effect on pain-related parameters in the cervical region. This result suggests that threshold and tolerance values obtained from the cervical region may be independent of TMD. However, this finding contrasts with those reported in previous studies^10,12–14^. These studies reported that the pain pressure threshold was lower in individuals with TMD compared to controls. In the present study, most individuals had mild jaw pain. This may have limited the potential for significant alterations in cervical pain thresholds and tolerance.

Finally, TS from the cervical region was evaluated in this study, and TMD did not appear to influence this parameter. TS is commonly used to assess central nervous system responsivity to nociceptive input^24^. Although research on endogenous pain modulation in myogenic TMD has expanded, consistent evidence demonstrating enhanced TS in this population is still lacking^25^. No group differences were observed in the present study. This finding suggests that TMD does not substantially affect TS in the cervical region. In the present study, TS was assessed using threshold-level stimuli rather than suprathreshold stimuli, which may reduce the robustness of TS responses. Future studies comparing distinct TMD subtypes—such as myogenic, disc displacement, and arthrogenic forms—with suprathreshold stimuli may provide further insight into potential differences in pain modulation across clinical presentations.

This study has some limitations. Firstly, although all participants in the TMD group were diagnosed using DC/TMD criteria, the presence of different TMD etiologies within the sample represents a methodological limitation. Secondly, the severity of TMD dysfunction among participants was predominantly mild to moderate, which may not fully capture the potential effects of more severe cases of TMD on NA, CSP, and pain-related parameters. Additionally, during the joint position error assessments, the order of testing (right rotation followed by left rotation) was not randomized. This fixed sequence may have introduced a potential order effect; therefore, the lack of test randomization should be considered a methodological limitation. Moreover, jaw pain was assessed only through pressure pain threshold, and other pain characteristics were not measured or included in the analyses. And also the cross-sectional design of the study limits the ability to infer causality between TMD and changes in NA. Longitudinal studies are necessary to better understand the temporal relationship between these variables. Finally, the study only included young adults aged 18–25 years, which limits the applicability of the results to other age groups. Future research should include a more diverse age range, focus on evaluating cervical outcomes within specific TMD subtypes to clarify potential subtype-related differences, and further explore the effects of TMD across different populations.

One of the key strengths of the present study is that it is the first to comprehensively examine the effects of TMD on NA and CSP. The present study showed that TMD led to significant changes primarily in body awareness rather than in somatosensory performance. This valuable finding makes a significant contribution to the literature by enhancing our understanding of the impact of TMD, and body awareness, which should be considered in clinical assessments.

Another notable aspect of this study is the separate evaluation of joint position errors in both the global cervical and isolated upper cervical regions. To the best of our knowledge, no previous study has examined CSP with this level of detail. This multidimensional approach offers a unique contribution to the literature.

## CONCLUSION

This study provided new insights into the impact of TMD on NA, CSP, and pain sensitivity. Although no differences were found between the groups in cervical joint position error tests or pain sensitivity, the findings underscored the importance of considering body awareness in clinical assessments. This study also pioneered the comprehensive evaluation of both global and isolated upper CSP, filling an important gap in the literature. Future research should explore these effects across different severities and diagnostic subtypes to enhance diagnostic and treatment strategies.

## Data Availability

The datasets generated and/or analyzed during the current study are available from the corresponding author upon reasonable request.
